# CD40 in Clinical Inflammation: From Multiple Sclerosis to Atherosclerosis

**DOI:** 10.1155/1998/69628

**Published:** 1998

**Authors:** Jon D. Laman, Mark De Boer, Bert A. 'T Hart

**Affiliations:** 1 Division of Immunological and Infectious Diseases TNO Prevention and Health (TNO-PG) PO Box 2215 Leiden 2301 CE The Netherlands; 2 Department of Immunology Erasmus University Rotterdam The Netherlands; 3 panGenetics BV Amsterdam The Netherlands; 4 Department of Immunobiology Biomedical Primate Research Center (BPRC) Rijswijk The Netherlands

**Keywords:** Macrophages, immunocytochemistry, accessory molecules, costimulation

## Abstract

The interactions of CD40 and CD40L have been known for some time to critically regulate B-cell responses with respect to proliferation, isotype switching, antibody production, and memory formation. More recent findings demonstrated that CD40 can be expressed on several other antigen-presenting cell (APC) types such as macrophages, dendritic cells, and fibroblasts. This expression of CD40 regulates T-cell-APC interaction and is centrally involved in a wide array of inflammatory events. Here, currently available data are reviewed demonstrating that CD40- CD40L interactions are operational in two chronic inflammatory clinical conditions, namely, multiple sclerosis and atherosclerosis. The functional correlates of these interactions are discussed in the light of recent other findings, shedding light on the multiple effects of CD40- CD40L interactions.


*Developmental Immunology*, 1998, Vol. 6, pp. 215–222
Reprints available directly from the publisher
Photocopying permitted by license only

© 1998 OPA (Overseas Publishers Association)
N.V. Published by license under the
Harwood Academic Publishers imprint,
part of the Gordon and Breach Publishing Group
Printed in Malaysia

*Minireview*

### CD40 in Clinical Inflammation: From Multiple Sclerosis to Atherosclerosis

JON D. LAMAN[a*], MARK DE BOER[b] and BERT A. 'T HART[c]

[a]*Division of Immunological and Infectious Diseases, TNO Prevention and Health (TNO-PG), PO Box 2215, 2301 CE Leiden and Department of Immunology, Erasmus University Rotterdam, The Netherlands*; [b]*PanGenetics BV, Amsterdam, The Netherlands*; [c]*Department of Immunobiology, Biomedical Primate Research Center (BPRC), Rijswijk, The Netherlands*

(*Received 6 November 1996; In final form 20 April 1997*; Accepted 12 August 1997)

The interactions of CD40 and CD40L have been known for some time to critically regulate B-cell responses with respect to proliferation, isotype switching, antibody production, and memory formation. More recent findings demonstrated that CD40 can be expressed on several other antigen-presenting cell (APC) types such as macrophages, dendritic cells, and fibroblasts. This expression of CD40 regulates T-cell-APC interaction and is centrally involved in a wide array of inflammatory events. Here, currently available data are reviewed demonstrating that CD40-CD40L interactions are operational in two chronic inflammatory clinical conditions, namely, multiple sclerosis and atherosclerosis. The functional correlates of these interactions are discussed in the light of recent other findings, shedding light on the multiple effects of CD40-CD40L interactions.

*Keywords:* Macrophages, immunocytochemistry, accessory molecules, costimulation

### MULTIPLE ROLES OF CD40-CD40 LIGAND INTERACTIONS

The past 4 years have seen such a flurry of papers on the multiple and diverse roles of the CD40-CD40L receptor pair (reviewed in detail by Laman et al., 1996; Van Kooten and Banchereau, 1996) that the interaction of these two molecules has recently been likened to a "master regulator" of the immune system (Grewal and Flavell, 1996). Initially, studies focused on elucidation of the role of CD40L transiently expressed on activated T cells in proliferation, differentiation, and function of CD40-positive B cells. Signals through CD40 trigger a wide array of B-cell activities, such as increased expression of MHC class II, CD23, CD25, CD69, and CD44, transition of LFA-1 to the high-affinity state, proliferation, locomotion, and homotypic adhesion. In conjunction with appropriate cytokines, CD40 ligation leads to antibody production, isotype switching, and direction of B cells

*Corresponding author.

into the memory pathway. The indispensable role of CD40 ligation and the nonredundancy in CD40L function is highlighted by the X-linked hyper IgM syndrome, in which patients lack isotypes other than IgM and suffer from recurrent infections (see preceding reviews).

More recently, it has become clear that functions of the CD40-CD40L receptor pair are not just limited to T cells signaling B cells. CD40 can be expressed on a wide array of APC, including B cells, follicular dendritic cells, (interdigitating) dendritic cells, fibroblasts, endothelial cells, and macrophages. This expression on most or all types of APC strongly suggests that CD40 signals are essential components of antigen presentation. In this respect, CD40L ligation has been shown to transduce signals required for T-cell priming (reviewed by Grewal and Flavell, 1996). However, this notion has been challenged by findings that some antiviral functions of $CD4^+$ T cells can be primed in a CD40-deficient environment (Oxenius et al., 1996). Another aspect of T-cell function is the finding that thymic epithelium expresses CD40, and that CD40L is involved in thymic selection of T cells (Foy et al., 1995). Antibody against CD40L can also inhibit the development of murine AIDS (Green et al., 1996), a viral disease targeting B cells. In addition, CD40L is required for an effective antiviral memory $CD8^+$ T-cell response (Borrow et al., 1996). With respect to autoimmune disease, anti-CD40L can interfere with disease development in mouse models for arthritis (Durie et al., 1993), systemic lupus erythematosus (Mohan et al., 1995; Early et al., 1996), and with disease in a mouse model for multiple sclerosis (MS) (Gerritse et al., 1996), as discussed in detail in what follows.

After the landmark paper of Alderson et al. (1993), which identified functional expression of CD40 on monocytes, some of the attention has shifted from B cells to effector functions of macrophages that are triggered through CD40 ligation, and that contribute to antigen-specific responses and inflammation. The different roles of CD40 in inflammation have been comprehensively reviewed by Stout and Suttles (1996),b. Some examples are the protective effector functions of macrophages triggered by CD40 ligation for immunity against *Pneumocystis carinii* (Wiley and Harmsen, 1995) and Leishmania species (Campbell et al., 1996; Kamanaka et al., 1996; Soong et al., 1996).

In what follows, we review data that we have obtained recently in two types of clinical chronic inflammation, namely, multiple sclerosis (MS), including the animal model for this disease, experimental autoimmune encephalomyelitis (EAE), and atherosclerosis. We then discuss how these data fit the currently known functional outcomes of interaction between CD40 and CD40L for distinct cell types.

### CD40 IN MULTIPLE SCLEROSIS AND EAE

### CD40 Is Expressed in the Central Nervous System in MS and EAE

In view of the known roles of CD40-CD40L mediating interactions between T cells and APC as well as T cells and B cells, we have previously evaluated this interaction in EAE in SJL mice. This forms a model for MS, a chronic inflammatory disease of the central nervous system (CNS). EAE induced in SJL mice by immunization with a peptide of the proteolipid protein ($PLP_{139-151}$) could be blocked by *in vivo* treatment with a monoclonal antibody against CD40L (Gerritse et al., 1996). Development of disease could be effectively blocked when antibody was coadministered with immunization for disease induction, but also when treatment was started several days after immunization. However, treatment was not effective during the later stages of established disease. These data argued that CD40-CD40L interactions are crucial for development of EAE, and that they operate at some point prior to manifestation of clinical signs.

To confirm a role of CD40-CD40L interactions in MS, we performed immunocytochemistry on frozen sections from autopsy material. Both abundant expression of CD40 and limited expression of CD40L could be demonstrated within perivascular inflammatory infiltrates of mononuclear cells *in situ* (Gerritse et al., 1996). Double-staining experiments for membrane expression of CD40 and intracytoplasmic activity of acid phosphatase showed that the large majority of cells expressing CD40 within inflammatory infiltrates belonged to the monocytic lineage. However, a minor fraction of the cells locally expressing CD40 could be identified as B cells as they also contained immunoglobulins (Gerritse et al., 1996). Currently, we are addressing the mechanisms by which anti-CD40L antibody treatment prevents disease in mice. In these experiments, we have found that CD40 is also expressed in the spinal cord of mice with EAE (Laman et al., manuscript in preparation). Using CD40L-deficient mice carrying a transgenic T-cell receptor for myelin basic protein (MBP), Grewal et al. (1996) confirmed our previous observations in anti-CD40L-treated mice, and extended these findings by showing that adoptive transfer of B7-1$^+$ APC could overcome the defect and supported production of IFN-y and development of EAE. Collectively, the data in mice and humans support the view that CD40-CD40L interactions are essential in these chronic inflammatory conditions and imply that novel therapeutic strategies could be developed with this in mind.

With the aim to develop animal models applicable to evaluate therapeutics suitable for use in humans, we analyzed expression of CD40, CD40L, B7-1, B7-2, and a panel of pro- and anti-inflammatory cytokines in the nonhuman primate marmoset species (*Callithrix jacchus*) (Laman et al., in press, b). Five marmosets were immunized with myelin purified from human white matter and suffered from attacks of acute EAE, corroborating earlier findings (Massacesi et al., 1995). Similar to the situation in MS, high levels of expression of CD40 were found within perivascular and periventricular infiltrates of mononuclear cells in the brain during active disease. Double staining for CD40 and acid phosphatase as described before revealed that, as in humans, many macrophages present within infiltrates express CD40. However, cells single positive for CD40 were also present, which probably are B cells or immature macrophages lacking acid phosphatase activity. In addition, only a proportion of locally present macrophages/activated microglia express CD40. Analysis of the anatomically restricted expression of CD40 may provide further clues to the functions of this molecule in chronic inflammation of the CNS.

### Possible Roles of CD40-CD40L Interactions in MS and EAE

We argue that CD40L expressed on activated T cells may have functional implications in MS and EAE at the following levels: transmigration of T cells through the (inflamed) endothelium of the blood-brain barrier and brain parenchyma; T-B-cell interaction leading to production of specific antibody within the CNS; priming of T cells and cytokine production; direct lytic effects of T cells, and finally T-cell macrophage interaction leading to activation of several different macrophage effector functions. These issues are discussed briefly in what follows.

CD40 is expressed by inflamed endothelium (e.g., Hollenbaugh et al., 1995; Karmann et al., 1995), and ligation leads to increased expression of several adhesion molecules (see Table I), thereby improving extravasation of mononuclear cells. CD40-CD40L interaction can mediate production of metalloproteinase 1 (MMP-1: collagenase) by T cells, which may improve transmigration of T cells (Dollery et al., 1995; Miltenburg et al., 1995).

Signals through CD40L are required for T-cell priming in several experimental settings (Grewal et al., 1995; Van Essen et al., 1995; reviewed by Grewal and Flavell, 1996), but not others (Oxenius et al., 1996). Expression of CD40 on APC either in the periphery or within mononuclear cell infiltrates (on macrophages) of the CNS in MS and EAE may serve this purpose. Peng et al. (1996) have shown that triggering T cells through CD40L leads to production of both Th1 and Th2 cytokines *in vitro* (see Table 1). A study by Vergelli et al. (1996) demonstrated that a novel population of CD4$^+$CD56$^+$ myelin-reactive T cells lyses target cells expressing CD56/neural cell adhesion molecule. It will be of interest to determine whether these cells express CD40L and whether ligation influences lytic activity.

Autoantibodies contribute to development of MS and EAE. T-B-cell interaction guided by CD40-

TABLE I Functions of CD40-CD40L Interactions in Relation to Multiple Sclerosis and Atherosclerosis

| |
|---|
| Antigen presentation: |
| Increased expression of ICAM-1, MHC class II, B7-2 (CD86), and CD40 on macrophages |
| T-cell priming |
| Antibody production: |
| B-cell proliferation |
| Increased expression of CD23, CD25, CD69, and CD44 |
| Isotype switching |
| Antibody production |
| Production of cytokines and other soluble factors: |
| Macrophages: IL-1, IL-6, IL-8, IL-12, TNF-$\alpha$, and NO by macrophages |
| T cells: IL-2, IL-4, IL-5, and IL-10 by T cells |
| Metalloproteinases: MMP-1/collagenase by T cells and MMP-9/gelatinase B by macrophages |
| Fibroblasts: IL-6 |
| Transmigration of mononuclear cells: |
| Expression of CD40 on inflamed endothelium |
| Increased expression of E-selectin, VCAM-1, and ICAM-1 after CD40 ligation |
| Tissue restructuring: |
| Proliferation of fibroblasts |
| Increased expression of ICAM-1 and VCAM-1 by fibroblasts |

CD40L is required for the thymus-dependent antibody response against protein components of the myelin sheath such as MBP and PLP. It was shown previously that antibody forming cells against MBP are present within mononuclear cell infiltrates in MS tissue (Gerritse et al., 1993, 1994).

Macrophages can potentially contribute to myelin loss and local inflammation in many ways, including (auto)-antigen presentation, production of cytokines and chemokines, proteolytic enzymes, nitric oxide, reactive oxygen species, and lipid peroxidation. *In vivo* depletion studies of macrophages have confirmed the requirement for this cell type in development of EAE (Huitinga et al., 1995). In addition, transgenic mice expressing the macrophage/microglia cytokine gene IL-3 in astrocytes show infiltration of macrophages but not lymphocytes, in conjunction with primary demyelination and motor deficits (Chiang et al., 1996). The role of macrophages is further supported by the finding that neutralization of the macrophage inflammatory protein-$\alpha$ (MIP-1$\alpha$) inhibits disease (Karpus et al., 1995).

Macrophage functions relevant to MS and EAE that can be triggered through CD40 include improved APC function through upregulation of ICAM-1, MHC class II, CD86, and CD40 itself (Kiener et al., 1995); production of proinflammatory cytokines (TNF-$\alpha$, IL-1$\beta$, IL-6, IL-8, and IL-12p40) (Alderson et al., 1993; Wagner et al., 1994; Kiener et al., 1995; Shu et al., 1995; Kato et al., 1996) (see also Table I). Probert et al. (1995) have shown that a transgenic mouse strain expressing TNF-$\alpha$ in the CNS has macrophage infiltration, demyelination, and severe neurological disease. The role of IL-12, a typical Th1 proinflammatory cytokine (reviewed by Seder et al., 1996), deserves special attention in the light of CD40-CD40L interactions. IL-12 promotes the development of EAE (Leonard et al., 1995), probably by stimulating the development of naive $CD4^+$ T cells into Th1 cells producing IFN-$\gamma$, but not IL-4. Shu et al. (1995) have argued that antigen presentation by monocytes may favor persistence of Th1 responses as production of IL-12 by monocytes can in turn provoke INF-$\gamma$ production by Th1 and Th0 cells. In support of this, Stuber et al. (1996) have shown that the same antibody we used to prevent EAE in mice (Gerritse et al., 1996) inhibits IL-12 production and thereby may prevent priming of T cells. Interestingly, two recent papers have shown that mouse dendritic cells produced high levels of IL-12 in response to CD40L, even in the absence of cognate antigen recognition (Cella et al., 1996; Koch et al., 1996). In addition, links exist between CD40L expression and production of IL-12 and NO, both in EAE (Waldburger et al., 1995) and in cell-mediated immunity against *Pneumocystis carinii* (Wiley and Harmsen, 1995) and

Leishmania species (Campbell et al., 1996; Kamanaka et al., 1996; Soong et al., 1996).

### CD40 IN ATHEROSCLEROSIS

### CD40 Is Expressed in Atherosclerotic Lesions

In view of our previous findings in MS and EAE, we considered the possibility that CD40-CD40L interactions are involved in aspects of atherosclerosis as well (Laman et al., 1997). This disease results from an extremely complex and only partly understood interplay among diverse components such as cholesterol metabolism, hemodynamic stress, the blood coagulation system, and risk factors such as obesity and smoking. The immune system is critically involved in atherosclerosis, as evidenced by high numbers of activated T cells and macrophages within atherosclerotic plaques (Hansson and Libby, 1996; Raines et al., 1996), and the involvement of mononuclear cells in production of cytokines and growth factors (Libby and Ross, 1996).

To assess the hypothesis that CD40-CD40L interactions play a role in atherosclerosis, we analyzed the expression of CD40 by means of immunohistochemistry on frozen sections from human plaque material. Atherosclerosis patients were surgically treated to remove plaques from the carotid artery (endarterectomy). CD40 was expressed in different regions of the plaque, and by cell types with distinct morphologies (Fig. 1c and 1d). To assess whether CD40 was expressed by macrophages within the plaque, double staining was performed for CD40 (blue in Figure 1d) and acid phosphatase, a lysosomal enzyme characteristic for macrophages (red in Fig. 1d). As double-staining cells were clearly present, we concluded that at least part of the CD40-expressing cells within plaques are macrophages.

A much wider analysis of the extent of CD40 (and CD40L) expression during atherosclerosis taking into account different clinical conditions, different locations and age of plaques, and anatomic sites within plaques now seems warranted as a basis to elucidate roles of CD40-CD40L interactions in this disease.

### Possible Roles of CD40-CD40L Interactions in Atherosclerosis

We argue that CD40-CD40L interactions could affect the atherosclerotic process at different levels, including transmigration of mononuclear cells into plaque areas; activation of T cells and cytokine production; activation of several macrophage effector functions; local antigen presentation; tissue restructuring through combined activities of T cells, macrophages, and fibroblasts.

The role of CD40 on endothelium, transmigration of cells, and production of cytokines by T cells has been addressed in the preceding section on EAE. The macrophage effector functions activated by CD40 ligation that may be operational in atherosclerotic plaques include adherence to CD40L-expressing cells, homotypic aggregation, and increased survival of cells, probably through protection from apoptosis Alderson et al., 1993). Furthermore, the antigen-presenting function can be improved through upregulation of accessory molecules (ICAM-1, MHC class II, B7-2, and CD40). With regard to bioactive compounds, CD40 ligation can costimulate NO production and secretion of the proinflammatory cytokines TNF-$\alpha$, IL-1$\beta$, IL-6, IL-8, and IL-12 (see Table 1 and references cited before). CD40L can also induce production of gelatinase B by monocytes and fibroblasts (Malik et al., 1996). Interestingly, by this mechanism, macrophages can digest collagen present in the fibrous cap of atherosclerotic plaques, and as such may contribute to fissure and release of the cap, leading to life-threatening occlusions of arteries (Galis et al., 1994). Fibroblasts express CD40 (Fries et al., 1995) and proliferate in response to CD40L signals (Yellin et al., 1995), suggesting that CD40-CD40L may act as a regulator of fibroblast expansion.

### CONCLUDING REMARKS

In this review, we have attempted to highlight the important similarities between expression and possibly function of CD40-CD40L in multiple sclerosis and EAE on the one hand, and atherosclerosis on data discussed here are derived from ongoing collaborations with Randy Noelle (Dartmouth Medical School, Lebanon, NH) and Bart de Smet (ICIN, Utrecht, the Netherlands). Marjan van Meurs and Arjan Schoneveld are gratefully acknowledged for expert immunocytochemistry.

### *References*

Alderson M.R., Armitage R.J., Tough T.W., Strockbine L., Fanslow W.C., and Spriggs M.K., (1993). CD40 expression by human monocytes: Regulation by cytokines and activation of monocytes by the ligand for CD40. J. Exp. Med. **178**: 669-674.

Armant M., Rubio M., Delespesse G., and Sarfati M. (1995). Soluble CD23 directly activates monocytes to contribute to the antigen-independent stimulation of resting T cells. J. Immunol. **155**: 4868-4875.

Borrow P., Tishon A., Lee S., Xu J., Grewal I.S., Oldstone M.B.A., and Flavell R.A. (1996). CD40L-deficient mice show deficits in antiviral immunity and have an impaired memory CD8+ CTL response. J. Exp. Med. **183**: 2129-2142.

Campbell K., Ovendale P.J., Kennedy M.K., Fanslow S.G., Reed S.G., and Maliszewski C.R. (1996). CD40 ligand is required for protective cell-mediated immunity to Leishmania major. Immunity **4**: 283-289.

Cella M., Scheidegger D., Palmer-Lehmann K., Lane P., Lanzavecchia A., and Alber G. (1996). Ligation of CD40 on dendritic cells triggers production of high levels of interleukin-12 and enhances T cell stimulatory capacity: T-T help via APC activation. J. Exp. Med. **184**: 747-752.

Chiang C-S., Powell H.C., Gold L.H., Samimi A., and Campbell L. (1996). Marcophage/microglial-mediated primary demyelination and motor disease induced by the central nervous system production of interleukin-3 in transgenic mice. J. Clin. Invest. **97**: 1512-1524.

Dollery C.M., McEwan J.R., and Henney A.M. (1995). Matrix metalloproteinases and cardiovascular disease. Circ. Res. **77**: 863-868.

Durie F.H., Fava R.A., Foy T.M., Aruffo A., Ledbetter J.A., and Noelle R.J. (1993). Prevention of collagen-induced arthritis with an antibody to gp39, the ligand for CD40. Science **261**: 1328-1330.

Early G.S., Zhao W., and Burns C.M. (1996). Anti-CD40 ligand antibody treatment prevents the development of lupus-like nephritis in a subset of New Zealand Black x New Zealand White mice. Response correlates with the absence of an anti-antibody response. J. Immunol. **157**: 3159-3164.

Foy T.M., Page D.M., Waldschmidt T.J., Schoneveld A., Laman J.D., Masters S.R., Tygrett L., Ledbetter J.A., Aruffo A., Claassen E., Xu J., Flavell R.A., Ohen S., Hedrick S.M., and Noelle R.J. (1995). An essential role for gp39, the ligand for CD40, in thymic selection. J. Exp. Med. **182**: 1377-1388.

Fries K.M., Sempowski G.D., Gaspari A.A., Blieden T., Looney R.J., and Phipps R.P. (1995). CD40 expression by human fibroblasts. Clin. Immunol. Immunopathol. **77**: 42-51.

Galis Z.S., Sukhova G.K., Lark M.W., and Libby P. (1994). Increased expression of matrix metalloproteinases and matrix degrading activity in vulnerable regions of human atherosclerotic plaques. J. Clin. Invest. **94**: 2493-2503.

Gerritse K., Deen C., Fasbender M., Ravid R., Boersma W.J.A., and Claassen E. (1994). The involvement of specific anti-myelin basic protein antibody-forming cells in multiple sclerosis immunopathology. J. Neuroimmunol. **49**: 153-159.

Gerritse K., Laman J.D., Noelle R.J., Aruffo A., Ledbetter J.A., Boersma W.J.A., and Claassen E. (1996). CD40-CD40 ligand interactions in experimental allergic encephalomyelitis and multiple sclerosis. Proc. Natl. Acad. Sci. USA **93**: 2499-2504.

Gerritse K., Slierendrecht B., Deen C., Fasbender M., Boersma W.J.A., and Claassen E. (1993). *In situ* detection of specific anti-myelin basic protein antibody forming cells with horseradish peroxidase myelin basic protein conjugates. EOS J. Immunol. Immunopharmacol. **13**: 63-65.

Green K.A., Crassi K.M., Laman J.D., Schoneveld A., Strawbridge R.R., Foy T.M., Noelle R.J., and Green W.R. (1996). Antibody to the ligand for CD40 (gp39) inhibits mouse AIDS (MAIDS) associated splenomegaly, hypergammaglobulinemia, and immunodeficiency in disease-susceptible C57BL/6 mice. J. Virol. **70**: 2569-2575.

Grewal I.S., and Flavell R.A. (1996). A central role of CD40 ligand in the regulation of CD4+ T cell responses. Immunol. Today **17**: 410-414.

Grewal I.S., Foellmer H.G., Grewal K.D., Xu J., Hardardottir F., Baron J.L., Janeway C.A., and Flavell R.A. (1996). Requirement for CD40 ligand in costimulation induction, T cell activation, and experimental allergic encephalomyelitis. Science **237**: 1864-1867.

Grewal I.S., Xu J., and Flavell R.A. (1995). Impairment of antigen-specific T cell priming in mice lacking CD40 ligand. Nature **378**: 617-620.

Hansson G.K. and Libby P. (1996). The role of the lymphocyte. In Atherosclerosis and Coronary Artery disease, Vol. 1, Fuster V., Ross R., and Topol E.J., Eds. (Philadelphia: Lippincott-Raven), pp. 557-568.

Hollenbaugh D., Mischel-Petty N., Edwards C.P., Simon J.C., Denfeld R.W., Kiener P.A., and Aruffo A. (1995). Expression of functional CD40 by vascular endothelial cells. J. Exp. Med. **182**: 33-40.

Huitinga I., Ruuls S.R., S Jung S., van Rooijen N., Hartung H.P., and Dijkstra C.D. (1995). Macrophages in T cell line mediated, demyelination and chronic relapsing experimental autoimmune encephalomyelitis in Lewis rats. Clin. Exp. Immunol. **100**: 344-351.

Kamanaka M., Yu P., Yasui T., Yoshida K., Kawabe T., Horii T., Kishimoto T., and Kishimoto H. (1996). Protective role of CD40 in Leishmania major infection at two distinct phases of cell-mediated immunity. Immunity **4**: 275-281.

Karmann K., Hughes C.C., Schechner J., Fanslow W.C., and Pober J.S. (1995). CD40 on human endothelial cells: Inducibility by cytokines and functional regulation of adhesion molecule expression. Proc. Natl. Acad. Sci. USA **92**: 4342-4346.

Karpus W.J., Lukacs N.W., McRae B.L., Strieter R.M., Kunkel S.L., and Miller S.D. (1995). An important role for the chemokine macrophage inflammatory protein-1$\alpha$ in the pathogenesis of the T cell-mediated autoimmune disease, experimental autoimmune encephalomyelitis. J. Immunol. **155**: 5003-5010.

Kato T., Hakamada R., Yamane H., and Nariuchi H. (1996). Induction of IL-12 p40 messenger RNA expression and IL-12 production of macrophages via CD40-CD40 ligand interaction. J. Immunol. **156**: 3932-3938.

Kiener P.A., Moran-Davis P., Rankin B.M., Wahl A.F., Aruffo A., and Hollenbaugh D. (1995). Stimulation of CD40 with purified soluble gp39 induces pro-inflammatory responses in human monocytes. J. Immunol. **155**: 4917-4925.

Koch F., Stanzl U., Jennewein P., Janke K., Heufler C., Kampgen E., Romani N., and Schuler G. (1996). High level IL-12 production by murine dendritic cells: Upregulation via MHC class II and CD40 molecules and downregulation by IL-4 and IL-10. J. Exp. Med. **184**: 743-746.

Laman J.D., Claassen E., and Noelle R.J. (1996). Functions of CD40 and its ligand, gp39. Crit. Rev. Immunol. **16**: 59-108.

Laman J.D., de Smet B.J.G.L., Schoneveld A., and van Meurs M. (In press, b). CD40-CD40L interactions in atherosclerosis. Immunol. Today.

Laman J.D., van Meurs M., Schellekens M.M., de Boer M., Melchers B., Massacesi L., Lassmann H., Claassen E., and 't Hart B.A. (In press, b). Involvement of accessory molecules and cytokines in experimental autoimmune encephalomyelitis (EAE) in marmoset monkeys (*Callithrix jacchus*). Am. J. Pathol.

Leonard J.P., Waldburger K.E., and Goldman S.J. (1995). Prevention of experimental autoimmune encephalomyelitis. J. Exp. Med. **181**: 381-386.

Libby P., and Ross R. (1996). Cytokines and growth regulatory molecules in atherosclerosis. In Atherosclerosis and Coronary Artery Disease, Vol. 1, Fuster V., Ross R. and Topol E.J., Eds. (Philadelphia: Lippincott-Raven), pp. 585-594.

Malik N., Greenfield B.W., Wahl A.F., and Kiener P.A. (1996). Activation of human monocytes through CD40 induces matrix metall oproteinases. J. Immunol. **156**: 3952-3960.

Massacesi L., Genain C.P., Lee-Parritz D., Letvin N.L., Canfield D., and Hauser S.L. (1995). Active and passively induced experimental autoimmune encephalomyelitis in common marmosets: A new model for multiple sclerosis. Ann. Neurol. **37**: 519-530.

Miltenburg A.M.M., Lacraz S., Welgus H.G., and Dayer J.-M. (1995). Immobilized anti-CD3 antibody activates T cell clones to induce the production of interstitial collagenase, but not tissue inhibitor of metalloproteinases, in monocytic THP-1 cells and dermal fibroblasts. J. Immunol. **154**: 2655-2667.

Mohan C., Shi Y., Laman J.D., and Datta S.K. (1995). Interaction between CD40 and its ligand gp39 in the development of murine lupus nephritis. J. Immunol. **154**: 1470-1480.

Oxenius A., Campbell K.A., Maliszewski C.R., Kishimoto T., Kikutani H., Hengartner H., Zinkernagel R.M., and Bachmann M.F. (1996). CD40-CD40 ligand interactions are critical in T-B cooperation but not for other anti-viral CD4 + T cell functions. J. Exp. Med. **183**: 2209-2218.

Peng X., Kasran A., Warmerdam P.A.M., de Boer M., and Ceuppens J.L. (1996). Accessory signaling by CD40 for T cell activation: Induction of Th1 and Th2 cytokines and synergy with interleukin-12 for interferon-$\gamma$ production. Eur. J. Immunol. **26**: 1621-1627.

Probert L., Akassoglou K., Pasparakis M., Kontogeorgos G., and Kollias G. (1995). Spontaneous inflammatory demyelinating disease in transgenic mice showing central nervous system-specific expression of tumor necrosis factor $\alpha$. Proc. Natl. Acad. Sci. USA **92**: 11294-11298.

Raines E.W., Rosenfield M.E., and Ross R. (1996). The role of macrophages. In Atherosclerosis and Coronary Artery Disease, Vol. 1, Fuster V., Ross R. and Topol E.J., Eds. (Philadelphia: Lippincott-Raven), pp. 539-555.

Seder R.A., Kelsall B.L., and Jankovic D. (1996). Differential roles for IL-12 in the maintenance of immune responses in infectious versus autoimmune disease. J. Immunol. **157**: 2745-2748.

Shu U., Kiniwa M., Wu C.Y., Maliszewski C., Vezzio N., Hakimi J., Gately M., and Delespesse G. (1995). Activated T cells induce interleukin-12 production by monocytes via CD40-CD40 ligand interaction. Eur. J. Immunol. **25**: 1125-1128.

Soong L., Xu J., Grewal I.S., Kima P., Sun J., Longley B.J., Ruddle N.H., McMahon-Pratt D., and Flavell R.A. (1996). Disruption of CD40-CD40 ligand interactions results in an enhanced suscep tibility to Leishmania amazonensis infection. Immunity **4**: 263-273.

Stout R.D., and Suttles J. (1996). The many roles of CD40 in cell-mediated inflammatory responses. Immunol. Today **17**: 487-492.

Stout R.D., Suttles J., Xu J., Grewal I.S., and Flavell R.A. (1996). Impaired T cell macrophage activation in CD40 ligand-deficient mice. J. Immunol. **156**: 8-11.

Stuber E., Strober W., and Neurath M. (1996). Blocking the CD40L-CD40 interaction in vivo specifically prevents the priming of T helper 1 cells through the inhibition of interleukin 12 secretion. J. Exp. Med. **183**: 693-698.

Van Essen D., Kikutani H., and Gray D. (1995). CD40 ligand-transduced co-stimulation of T cells in the development of helper function. Nature **378**: 620-623.

Van Kooten C., and Banchereau J. (1996). CD40-CD40 ligand, a multifunctional receptor-ligand pair. Adv. Immunol. **61**: 1-77.

Vergelli M., Le H., van Noort J.M., Dhib-Jalbut S., McFarland H., and Martin R. (1996). A novel population of CD4 + CD56 + myelin-reactive T cells lyses target cells expressing CD56/neural cell adhesion molecules. J. Immunol. **157**: 679-688.

Wagner D.H., Stout R.D., and Suttles J. (1994). Role of the CD40-CD40 ligand interaction in CD4+ T cell contact-dependent activation of monocyte interleukin-1 synthesis. Eur. J. Immunol. **24**: 3148-3154.

Waldburger K.E., Hastings R.C., Schaub R.G., Goldman S.J., and Leonard J.P. (1995). Adoptive transfer of experimental allergic encephalomyelitis after in vitro treatment with recombinant murine interleukin 12. Amer. J. Pathol. **148**: 375-382.

Wiley J.A., and Harmsen A.G. (1995). CD40 ligand is required for resolution of *Pneumocystis carinii* pneumonia in mice. J. Immunol. **155**: 3525-3529.

Yellin M.J., Winikoff S., Fortune S.M., Baum D., Lederman S., and Chess L. (1995). Ligation of CD40 on fibroblasts induces CD54 (ICAM-1) and CD106 (VCAM-1) up-regulation and IL-6 production and proliferation. J. Leukoc. Biol. **58**: 209-216.

